# The “Fracking” technique: a novel approach to crack deep calcified plaque in the common femoral artery with hydraulic pressure

**DOI:** 10.1186/s42155-021-00258-y

**Published:** 2021-09-27

**Authors:** Takuya Haraguchi, Tsutomu Fujita, Yoshifumi Kashima, Masanaga Tsujimoto, Tsuyoshi Takeuchi, Yutaka Tadano, Daisuke Hachinohe, Umihiko Kaneko, Ken Kobayashi, Daitaro Kanno, Katsuhiko Sato

**Affiliations:** Director of Cardiology and Head of Peripheral Artery Disease Center, Sapporo Heart Center, North 49, East 16, 8-1, Higashi ward, Sapporo, Hokkaido 007-0849 Japan

**Keywords:** Common femoral artery, Endovascular intervention, Calcified plaque, Atherectomy, Endarterectomy, Peripheral arterial disease, Balloon angioplasty, Intravascular lithoplasty, Intravascular ultrasound

## Abstract

**Background:**

The patency achieved by conventional peripheral interventions for atherosclerotic lesions in the common femoral artery (CFA), called the “no stenting zone”, is not superior to that achieved by surgical endarterectomy due to calcified plaque occupying the area. Plaque modification strategies to obtain acute gain in CFA patency provide the better clinical outcomes than standard balloon angioplasty. Atherectomy devices, which focus on the modification of superficial calcifications, contribute to the improvement of clinical outcomes. However, deep calcifications resist vessel expansion such that luminal gain is not easily achieved.

**Main text:**

We propose a novel calcified plaque modification technique, named the “fracking technique” (FT). The term fracking refers to how a rock is fractured by the high hydraulic pressure. In this technique, deep calcifications are cracked with hydraulic pressure via a balloon indeflator through an 18-gauge needle, which punctures calcifications to achieve greater acute luminal gain. Case 1 involved an 81-year-old male with eccentric calcified plaque in the right CFA. Conventional balloon angioplasty for the lesion yielded a suboptimal minimal lumen area (MLA), which increased from 6.2 to 10.7-mm^2^ on intravascular ultrasound (IVUS). The FT was implemented to obtain a larger MLA. After the FT was repeated at three locations at up to 8-atm, a greater MLA of 27.1-mm^2^ was achieved without complications. Case 2 involved a 72-year-old male undergoing hemodialysis due to diabetes mellitus who presented with ischemic pain in his right limbs at rest due to severe stenosis with eccentric calcification in the distal CFA. The MLA on IVUS before and after balloon angioplasty was 10.0-mm^2^ and 13.1-mm^2^, respectively, and this result was still suboptimal. The FT was attempted and successfully yielded a greater MLA of 28.9-mm^2^ without complications. Restenosis has not been detected for 2 years follow-up period.

**Conclusions:**

The FT is an effective option for treating calcified CFA lesions to achieve a larger lumen area. Long-term follow-up studies are necessary.

**Supplementary Information:**

The online version contains supplementary material available at 10.1186/s42155-021-00258-y.

## Background

Surgical treatment for lesions involving common femoral artery (CFA) is still considered the gold standard treatment because of its excellent long-term patency and limb salvage rates; however, the rates of the surgical complications, including perioperative death, are still unexpectedly high (Nguyen et al. 2015).

While peripheral interventions have become more common, conventional balloon angioplasty for atherosclerotic CFA lesions has failed to show promising results due to calcifications (Bonvini et al. 2011). Balloons, including drug-coated balloons, and atherectomy devices target superficial calcium. However, these devices cannot affect deep calcium, which resists vessel expansion and luminal gain (Dini et al. 2019). Additionally, atherectomy devices have a relatively high rate of complications such as vessel perforation and distal embolization (Finn et al. 2020).

## Main text

This is the first report of our novel technique, named the “fracking technique (FT)”, to modify deep calcium with hydraulic pressure through an 18-gauge needle, which punctures calcifications to crack them, achieve greater acute luminal gain, increase vessel compliance, and restore vessel mobility, serving as a new versatile treatment option for patients.

The process of FT is as follows. First, a 6-Fr guiding sheath is percutaneously inserted to establish the crossover system from the contralateral CFA. A 5000-IU of unfractionated heparin was injected intra-arterially from the guiding sheath for systemic heparization. After a guidewire crosses calcified CFA lesion, a balloon dilatation sized by intravascular ultrasound (IVUS) measurement for the lesion is performed. If unacceptable procedure results, of which are residual stenosis > 30% on angiography and insufficient acute gain on IVUS, remain after the balloon angioplasty, an 18-gauge needle (Terumo, Japan) without a plastic outer sheath is inserted into a part of the calcification that shows inadequate expansion on angiography and/or IVUS. Alternatively, a larger needle hole is not recommended to use, as it may cause hydraulic pressure leakage from the gap between the needle hole and calcified plaque. The balloon dilation is maintained during the FT to compress the lesion to make it denser and prevent dissection, which might extensively spread during the FT. After needle insertion, a 3.0-ml lock syringe is sequentially attached to the needle to confirm the location of the needle tip inside the plaque by injecting saline from the syringe. As the needle tip reaches dense areas of calcification and the plunger of the syringe cannot be pushed further, the location of the “fracking point” is determined. A balloon indeflator with a half concentration of contrast agent is connected to the needle, and the pressure of the indeflator is gradually increased in the area of dense calcification under angiography until a sudden drop in the pressure occurs, which indicates cracking of the calcification. This process is “fracking”. Fracking is repeatedly performed at several fracking points in calcified plaques until the fracking points cannot be detected anymore or a sufficient MLA is obtained. The contrast agent in the indeflator sometimes flows from the fracking point into the vessel lumen, which indicates that the deep calcification has been cracked and connected to the lumen. Often time the calcifications are cracked by less than 10-atm (atm) of pressure; however, a few severely calcified plaques may require up to 30-atm from our experiences. After fracking, dilatation using the previous balloon is performed to further compress the calcification, which has a network of grains, pores, and cracks due to fracking, this process also serves to stop bleeding at the fracking points. Usually, hemostasis can be achieved in less than 5 min. Finally, when IVUS demonstrates that a larger or the targeted MLA has been achieved, the procedure is finished. The typical process of the FT is shown in the supplementary material (Supplementary Movie 1).


**Additional file 1: Supplementary Movie 1.** The typical process of the fracking technique.


**Fig. 1:**
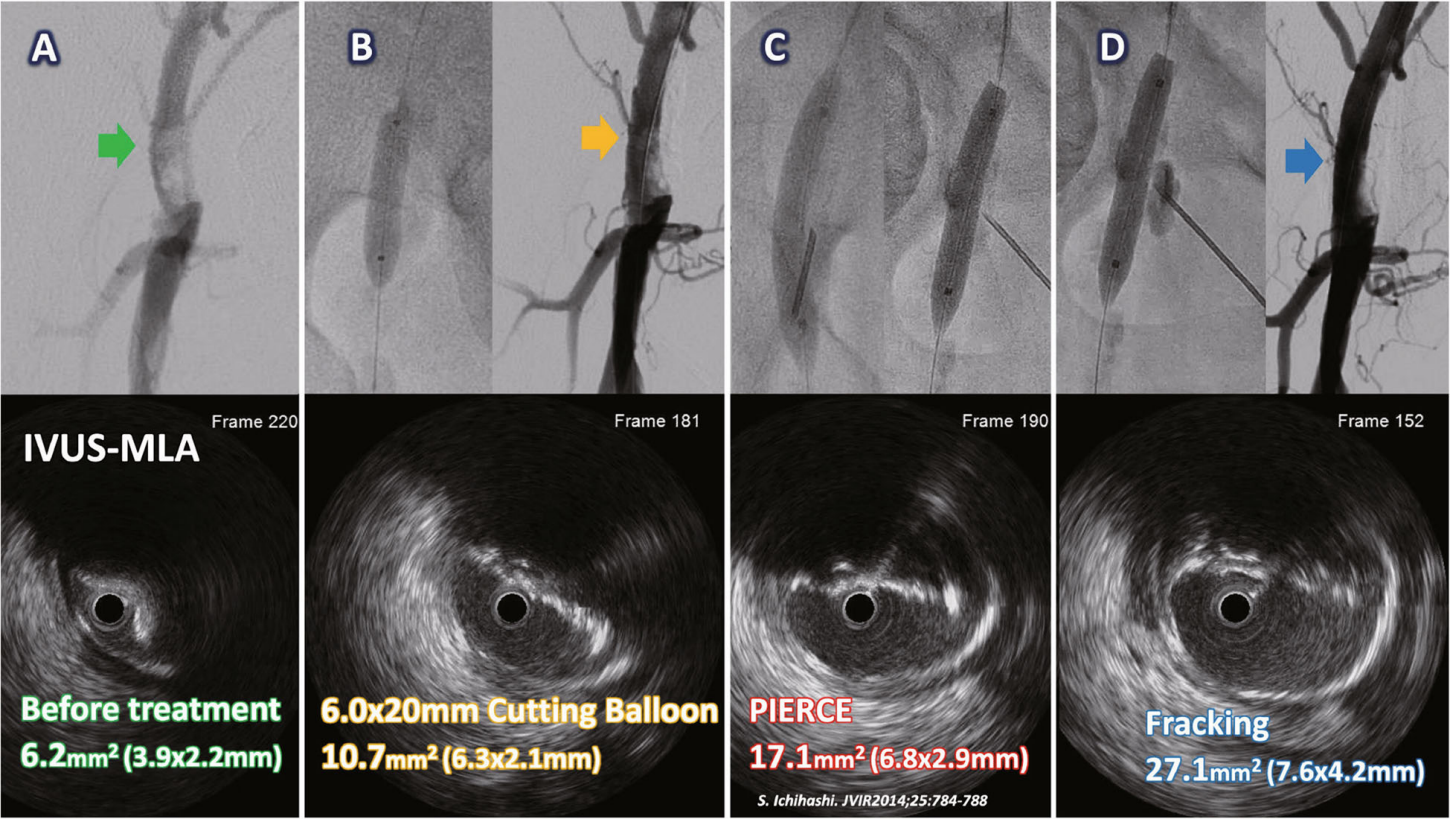
Angiography and intravascular ultrasound (IVUS) of the treatment with the fracking technique in case 1. **A** Control angiography for quantitative vessel analysis (QVA) showed stenosis of 94% in eccentric calcified plaque in the right common femoral artery (green arrow), and IVUS before treatment showed a minimal lumen area (MLA) of 6.2-mm^2^. **B** After dilatation with a 6.0 × 20-mm cutting balloon, QVA showed 24% stenosis (yellow arrow), and the MLA was 10.7-mm^2^. **C** After a 7.0 × 40-mm noncompliant balloon inflation and percutaneous direct needle puncture of calcified plaque (PIERCE) to modify the calcification (Ichihashi et al. 2014), the MLA was 17.1-mm^2^. **D** After the fracking was repeated at three locations at up to 8-atm, QVA showed an improvement of stenosis to 16% (blue arrow), and the MLA was significantly much greater, to 27.1-mm^2^

Here, we present the two representative cases of treatment with the FT. Case 1 involved an 81-year-old female with diabetes mellitus who presented with claudication in her right calf. A 6-Fr guiding sheath was inserted into the left CFA via a contralateral approach. Quantitative vessel analysis (QVA) demonstrated 94% stenosis with eccentric calcification, which occupied from the middle to distal part of the right CFA. A 0.014-in. guidewire passed through the lesion, and IVUS revealed eccentric calcification with an MLA of 6.2-mm^2^ (3.9 × 2.2-mm) (Fig. 1A). After dilation with a 6.0 × 20-mm cutting balloon, QVA showed 24% residual stenosis. However, the MLA (10.7-mm^2^; 6.3 × 2.1-mm) was not acceptable (Fig. 1B). The percutaneous direct needle puncture of calcified plaque (PIERCE) technique was attempted to modify the calcification (Ichihashi et al. 2014), and a 7.0 × 40-mm noncompliant balloon was used to dilate the lesion with maximal pressure. IVUS showed an improvement in MLA (17.1-mm^2^; 6.8 × 2.9-mm) (Fig. 1C). To further expand the MLA, the FT was implemented to modify the eccentric calcification, which could not be satisfactorily compressed with the conventional balloon angioplasty. After fracking was repeated at three locations at up to 8-atm, QVA showed significant improvement of the residual stenosis to 16% and an MLA of 27.1-mm^2^ (7.6 × 4.2-mm) (Fig. 1D). Finally, a satisfactory result was achieved without complications.

**Fig. 2:**
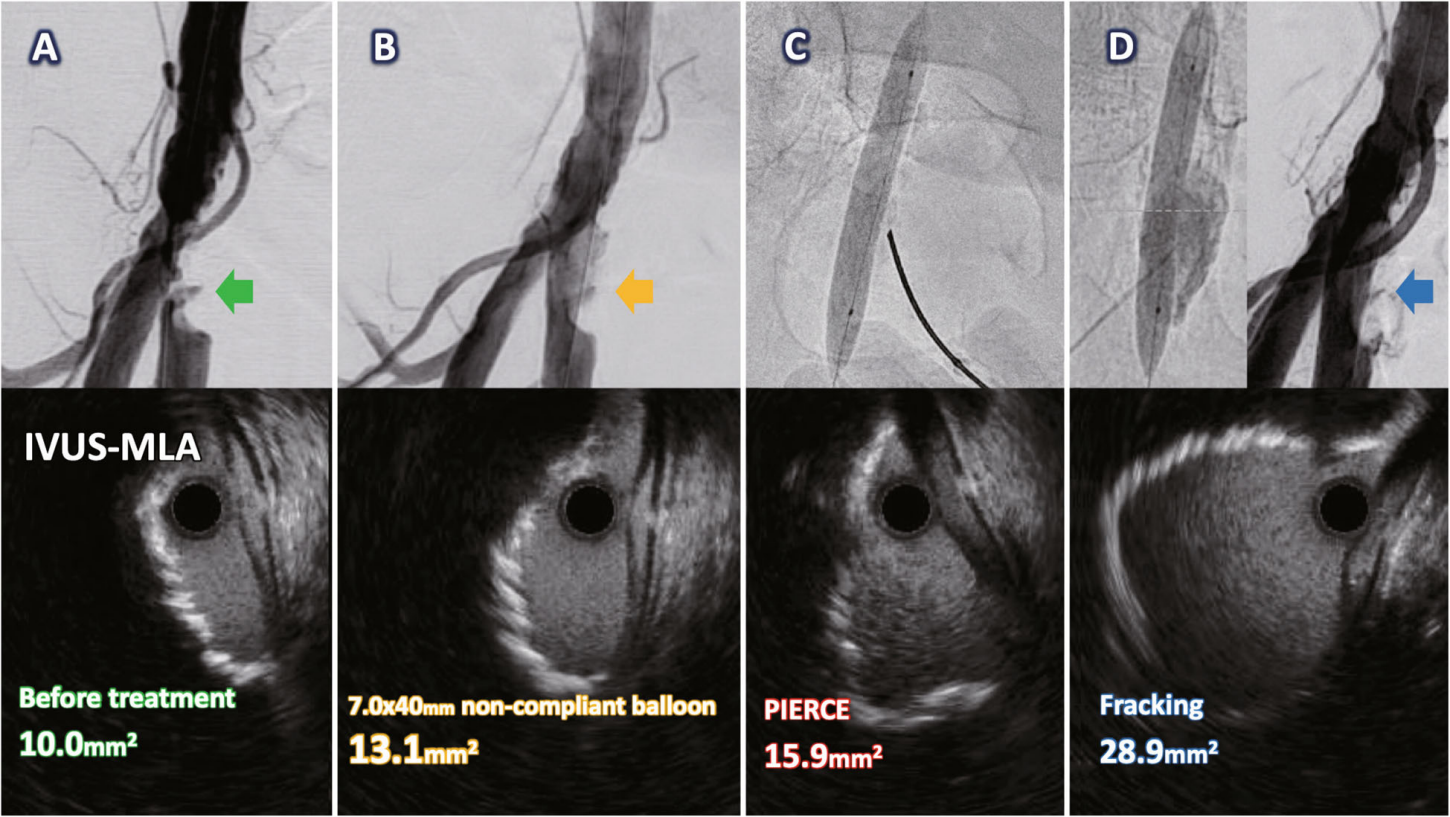
Angiography and intravascular ultrasound (IVUS) of the treatment with the fracking technique in case 2. **A** Control angiography for quantitative vessel analysis (QVA) showed eccentric calcified plaque in the distal common femoral artery with stenosis of 96% (green arrow), and IVUS before treatment showed a minimal lumen area (MLA) of 10.0-mm^2^. **B** After balloon dilatation with a 7.0 × 40-mm noncompliant balloon, QVA showed 42% stenosis (yellow arrow), and the MLA was 13.1-mm^2^. **C** After the 7.0 × 40-mm noncompliant balloon inflation and percutaneous direct needle puncture of calcified plaque (PIERCE) (Ichihashi et al. 2014), the MLA was unsatisfactory, at 15.9-mm^2^. **D** After the fracking technique was repeated at two locations at up to 5-atm, QVA showed an improvement in stenosis to 26% (blue arrow), and the MLA was significantly greater, at 28.9-mm^2^

Case 2 involved a 72-year-old male undergoing hemodialysis who presented with ischemic pain in his right limbs at rest. Angiography revealed severe stenosis with eccentric calcification in the distal CFA. The QVA and MLA on IVUS before and after dilation with a 7.0 × 40-mm noncompliant balloon was 96% and 10.0-mm^2^, 42% and 13.1-mm^2^, respectively (Fig. 2A, B). The MLA showed a slight improvement of 15.9-mm^2^ after PIERCE technique (Fig. 2C). To achieve the greater acute luminal gain, the FT was attempted at two locations at up to 5-atm, yielding a larger MLA of 28.9-mm^2^ without complications (Fig. 2D). ABI was normalized after the procedure, and these patients had lack of hematoma damage to CFA and absence of downstream trash embolization in perioperative period. Furthermore, restenosis caused by neointimal hyperplasia occurrence has not been detected for 2 years follow-up period.

## Discussion

The concept of the FT, which targets deep calcified plaques, is the same as that of intravascular lithoplasty (IVL) (Brodmann et al. 2019). IVL uses pulsatile sonic pressure waves to modify calcified lesions from the intimal to the medial area. Excimer laser atherectomy is also a unique procedure that modifies the morphology of the surface-plaque as well as the hard tissues underneath the calcification (Reekers et al. 1991). Conversely, the FT cracks calcified lesions from the outer to the middle area, with the advantage of manual control over damage to the surrounding tissues. The FT might have the potential to yield a larger MLA, similar to IVL. Notably, in our cases, these IVUS images demonstrated a larger MLA was achieved without changing the superficial shape of the calcification (Fig. 2A-D), and this phenomenon implies that the FT mainly fracture the deep calcification. Therefore, we assume a lower probability of distal embolization. Accordingly, distal protection device might not be mandatory. Long-term follow-up studies are necessary.

In terms of limitations, the FT can be performed in the infrainguinal arteries, especially the CFA, but not in the suprainguinal arteries. Calcifications on the dorsal side of the artery are also difficult to puncture with the needle. The effect of the FT depends on the volume and density of the calcification. The FT may be more effective for eccentric than concentric calcified lesions. Additionally, there is a learning curve involved in puncturing the calcification compressed with the balloon without puncturing the balloon or damaging the artery.

## Conclusions

The FT is an effective option for treating calcified CFA lesions when appropriate dilatations of procedural success are difficult to achieve. Whether a larger MLA by the FT might lead to long-term patency needs to be compared with conventional balloon angioplasty in large patient populations.

## Data Availability

The datasets used and/or analyzed during the current study are available from the corresponding author on reasonable request.

## Abbreviations

CFA: Common femoral artery
FT: Fracking technique
MLA: Minimal lumen area
IVUS: Intravascular ultrasound
atm: Atmospheres
QVA: Quantitative vessel analysis
PIERCE: Percutaneous direct needle puncture of calcified plaque
IVL: Intravascular lithoplasty
